# Brain stimulation with 40 Hz heterochromatic flicker extended beyond red, green, and blue

**DOI:** 10.1038/s41598-024-52679-z

**Published:** 2024-01-25

**Authors:** Mark Alexander Henney, Marcus Carstensen, Martin Thorning-Schmidt, Marta Kubińska, Manja Gersholm Grønberg, Mai Nguyen, Kristoffer Hougaard Madsen, Line Katrine Harder Clemmensen, Paul Michael Petersen

**Affiliations:** 1 Department of Applied Mathematics and Computer Science, Technichal University of Denmark, Kgs. Lyngby, 2800 Denmark; 2 OptoCeutics ApS, Copenhagen, 1610 Denmark; 3 Department of Electrical and Photonics Engineering, Technichal University of Denmark, Kgs. Lyngby, 2800 Denmark; 4 Danish Research Centre for Magnetic Resonance, Centre for Functional and Diagnostic Imaging and Research, Copenhagen University Hospital Amager and Hvidovre, Hvidovre, 2650 Denmark

**Keywords:** Biomedical engineering, Colour vision, Neurophysiology, Biophotonics

## Abstract

Alzheimer’s disease (AD) is associated with electrophysiological changes in the brain. Pre-clinical and early clinical trials have shown promising results for the possible therapy of AD with 40 Hz neurostimulation. The most notable findings used stroboscopic flicker, but this technique poses an inherent barrier for human applications due to its visible flickering and resulting high level of perceived discomfort. Therefore, alternative options should be investigated for entraining 40 Hz brain activity with light sources that appear less flickering. Previously, chromatic flicker based on red, green, and blue (RGB) have been studied in the context of brain-computer interfaces, but this is an incomplete representation of the colours in the visual spectrum. This study introduces a new kind of heterochromatic flicker based on spectral combinations of blue, cyan, green, lime, amber, and red (BCGLAR). These combinations are investigated by the steady-state visually evoked potential (SSVEP) response from the flicker with an aim of optimising the choice of 40 Hz light stimulation with spectrally similar colour combinations in BCGLAR space. Thirty healthy young volunteers were stimulated with heterochromatic flicker in an electroencephalography experiment with randomised complete block design. Responses were quantified as the 40 Hz signal-to-noise ratio and analysed using mixed linear models. The size of the SSVEP response to heterochromatic flicker is dependent on colour combinations and influenced by both visual and non-visual effects. The amber-red flicker combination evoked the highest SSVEP, and combinations that included blue and/or red consistently evoked higher SSVEP than combinations only with mid-spectrum colours. Including a colour from either extreme of the visual spectrum (blue and/or red) in at least one of the dyadic phases appears to be more important than choosing pairs of colours that are far from each other on the visual spectrum. Spectrally adjacent colour pairs appear less flickering to the perceiver, and thus the results motivate investigations into the limits for how alike the two phases can be and still evoke a 40 Hz response. Specifically, combining a colour on either extreme of the visual spectrum with another proximal colour might provide the best trade-off between flickering sensation and SSVEP magnitude.

## Introduction

Alzheimer’s disease (AD) is a neurodegenerative disorder projected to affect more than 66 million people worldwide and cost society 2 trillion dollars by 2030^1^. Patients, caregivers, and families are affected by the suffering associated with the late-stage consequences of cognitive decline.

With exception of the recently FDA-approved aducanemab and lecanemab, disease-modifying pharmaceutical therapies for AD have had limited success. The targets of these monoclonal antibodies are the hallmark Amyloid deposits in the brain that define AD. However, interest has increased for the interpretation of AD as a brain network dysfunction, in which the electrophysiological changes are studied as pathological markers and potential mechanisms of action for therapeutics^2–4^.

For more than 30 years, electrophysiologists have been aware of the relation between AD and 40 Hz brain oscillations^5^. The interest for inherent, induced, and evoked 40 Hz oscillatory neural potentials and their relation to cognition goes back even further. While there is some uncertainty as to what can be considered a “40 Hz oscillation”, several authors present early evidence of a resonant phenomenon in the mammalian visual cortex in the proximity of 40 Hz^6–15^.

Throughout the following decades and until recently, an expanding number of publications have described findings that support and elaborate on the importance of 40 Hz oscillations. Preattentive visual stimulation at 40 Hz but not other frequencies improved the reaction time in a target detection task^16^. Neural oscillators were then found to display preferences for 10, 20, 40, and 80 Hz frequencies when human sycnhronous oscillations were measured in response to stimulation frequencies of 1-100 Hz^17^. In 2009, key insights were gained in regards to the importance of fast-spiking parvalbumin-positive interneurons in the emergence and entrainment of gamma oscillations, predominantly in the 40 Hz range^18,19^.

Neural oscillation patterns differ significantly between healthy subjects and patients suffering from mild cognitive impairment (MCI) and AD^20^, and a general shift towards lower frequency oscillations and reduction of higher frequency oscillations is found in AD patients, compared to healthy controls^21^. The oscillatory synchrony in networks which support cognition is altered many years prior to the onset of clinical symptoms^22^. Furthermore, these alterations seem to predict future pathology and brain atrophy, and therapeutic strategies that aim to counter these abnormalities may be important in the progression and treatment of AD.

Neurostimulation at 40 Hz has been utilised as a potential therapeutic strategy for restoring the spectral aberrations of AD. In 2016, researchers began suggesting the use of gamma entrainment using sensory stimulation (GENUS) in the form of visual stimulation with 40 Hz stroboscopic flicker as a form of treatment for AD^23^. This GENUS system used white LEDs with a correlated colour temperature (CCT) of 4000 K, flickering at 40 Hz with a 50% duty cycle to provide visual stimulation. The nature of the temporal modulation is stroboscopic, i.e., flicker with 100% modulation depth. Throughout a wide range of experiments with validated mouse models of AD, in which the characteristics of the light have been kept constant, GENUS therapy shows promising results in regard to neuroprotective effects^23–29^. Human safety and feasibility trials have since proceeded with the same light settings in combination with 40 Hz clicking sounds^30^. Their reported study drop-out rate of 28% may, however, be indicative of an issue with stroboscopic stimulation paradigms.

Using stroboscopic flicker, also called temporally modulated light, poses an inherent barrier for treatment when used in human applications due to its flickering nature. The side effects of stroboscopic light diminish as the temporal modulation frequency increases, until the flicker is no longer perceptible^31^. The critical flicker-fusion frequency (CFF) is the point at which a temporally modulated light is no longer perceived as flickering and is dependent on the colour and modulation depth of the flicker as well as the observer. For the stroboscopic light at 100-500 lux^26^ that was used in the studies described in Refs.^23–29^, the CFF is in the range of 55-70 Hz^32^ and thus at least 15 Hz above the stimulation frequency of 40 Hz, rendering the flicker very visible to observers. This issue motivates the search for an alternative form of stimulation delivery with the same down-stream clinical efficacy as preliminary evidence attributes 40 Hz stroboscopic flicker. A 40 Hz light source with a CFF closer to or lower than 40 Hz would reduce the perceived flicker, and such a solution could improve treatment adherence and thus also ultimately effectiveness.

Heterochromatic (or simply chromatic) flicker substitutes the *off*-phase of stroboscopic flicker with a phase consisting of light with a different colour such that the light alternates between two different colours. According to^31^, replacing stroboscopic flicker with chromatic flicker may reduce the CFF to downwards of 15-30 Hz (depending on the luminance), improve neural oscillatory response, and reduce possible side effects. As the stimulation frequency supersedes the CFF, this makes chromatic flicker a possible candidate for a 40 Hz stimulation paradigm in humans.

**Figure 1:**
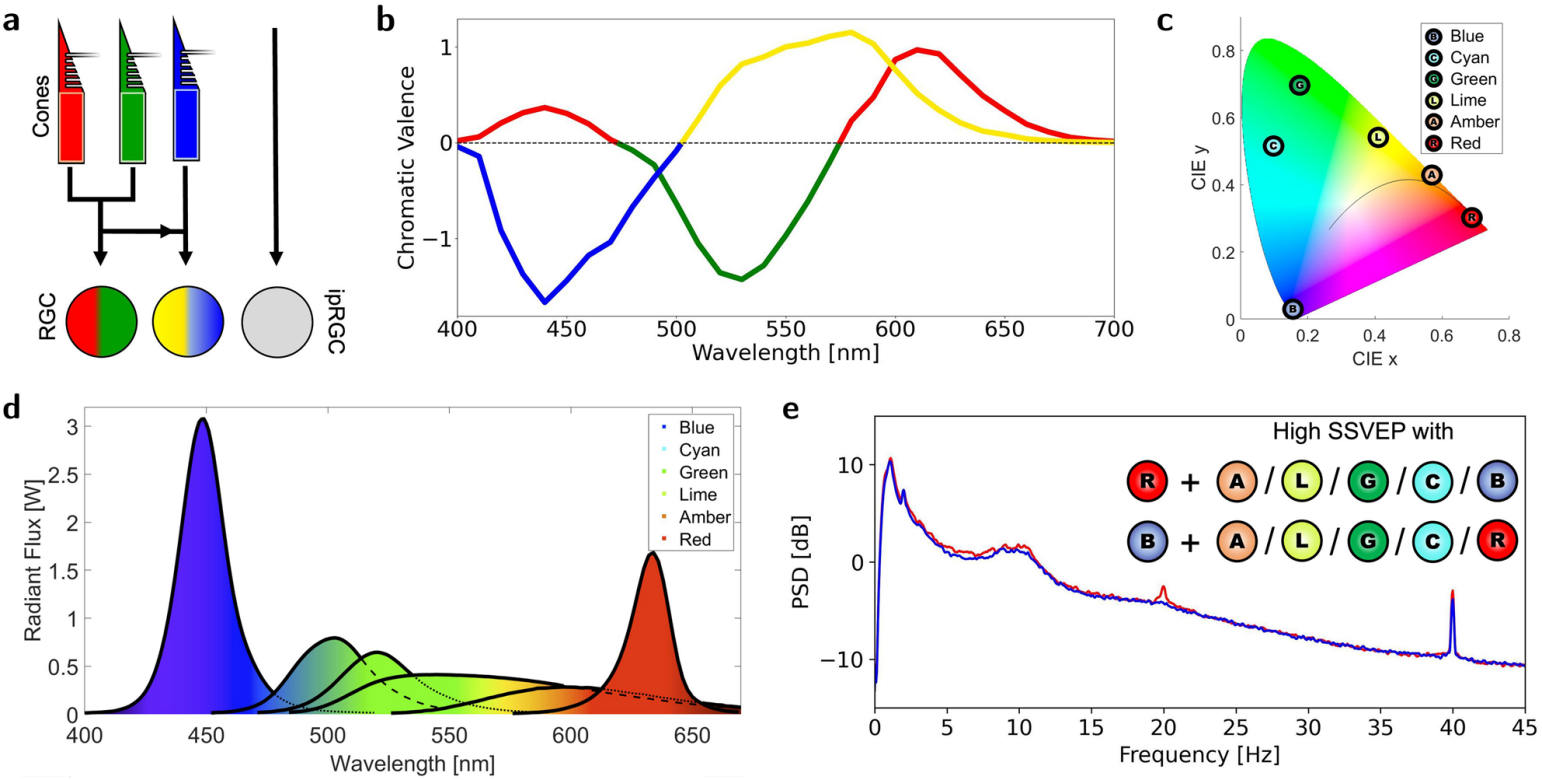
Heterochromatic Flicker Study Summary: The visual pathway from rods, cones, and retinal ganglion cells, through the lateral geniculate nucleus, to the visual cortex forms a complex pathway for human processing and experience of light and colour. In an electroencephalography experiment, we stimulate with 40 Hz heterochromatic flicker with the 15 dyadic combinations of six colours: Blue, cyan, green, lime, amber, and red. (**a**) Retinal processing of visual stimulation integrates both visual effects detected by cones and non-visual effects bypassing cones and detected directly by intrinsically photosensitive retinal ganglion cells (ipRGCs). (**b**) Colour opponency axes adapted from^35^, in which the chromatic valence indicates the relative sensitivity of each of the red-green and blue-yellow axes, respectively, to a given wavelength, and thus defines the perceived colour. (**c**) CIE coordinates plotted for the six LEDs (see also Fig. S.2 and Table S.1 of the supplementary material). (**d**) Spectra of the six LEDs (see also Fig. S.1 of the supplementary material). The LEDs are matched empirically by luminous flux to avoid luminance flicker, which leads to an increased radiant flux for blue and red to compensate for the lower sensitivity to these colours. (**e**) Representative average power spectra for high-response combinations. The highest steady-state visually evoked potentials were entrained when the flicker included red and/or blue, suggesting that inclusion of the upper or lower extreme of the visual spectrum is more important than choosing spectrally different colours. For heterochromatic flicker, colour opponency may explain why the sensation of flicker appears to be disjoint from the size of the SSVEP.

Steady-state visually evoked potentials (SSVEPs) are neural oscillatory responses that arise from and are phase-locked to an external temporally modulated visual stimulus. The SSVEP generated by visual stimuli is conveyed by the retinal ganglion cells (RGC). These cells process input from the photoreceptive cells (cones and rods) by forming a receptive field with inhibitory connections in the centre and excitatory connections in the surround, or vice versa^33^. Additionally, intrinsically photosensitive RGCs (ipRGCs) contribute to the projected signal through non-image forming irradiance information^34^ (see Fig. 1a).

The visual system of colour consists of two pathways, mediated by cones with variable sensitivity to photons of different wavelengths, and also called colour opponency axes^36^ (see Fig. 1b). The cones are typically referred to as short-wave (S), middle-wave (M), and long-wave (L) sensitive cones. The pathways are 1: A red-green chromatic pathway (R/G axis) and 2: A blue-yellow chromatic pathway (B/Y axis). They commence with the receptive field of the RGCs, which integrate inhibitory and excitatory action potentials from 1: L-cones relative to the M-cones, and 2: S-cones relative to the additive signal from the M- and L-cones. Perceivable flicker is thought to originate from the rods and the B/Y axis^37–42^. One consequence of this is that temporally modulated light alternating between two light sources of similar spectral colour is perceived to be less flickering than light having a large colour difference. Thus, for the purpose of limiting flicker, choosing spectrally similar colours in a heterochromatic flicker setup is advantageous.

While some have studied the SSVEP response to chromatic flicker in the context of brain-computer interfaces (BCI), these have focused mainly on combinations of red, green, and blue (RGB) colours^43–48^. To augment the prior research with chromatic flicker using combinations of RGB, we propose to extend the colour space by using blue, cyan, green, lime, amber, and red (BCGLAR). These colour combinations provide a wider array of options to study the colour-dependency aspect of the SSVEP and were chosen to better cover the International Commission on Illumination (CIE) 1931 colour space (see Fig. 1c and Fig. 1d). To the knowledge of the authors, no studies have investigated potential therapeutic effects of chromatic flicker for AD with the possible exception of^49^ which used chromatic flicker with dyades of almost metameric light (described in^50,51^) rather than monochromatic light. Despite the vast advances of pre-clinical and clinical use of 40 Hz stroboscopic flicker, the opportunities of chromatic flicker are still largely unknown.

In this study, summarised in Fig. 1, we examined the acute neural oscillatory responses to various types of chromatic flicker using 15 combinations of colours from the BCGLAR colour space. This may present new candidates for 40 Hz visual stimulation therapies with reduced flicker sensations compared to stroboscopic flicker, especially if spectrally similar colour combinations such as red and amber can evoke high SSVEPs (see Fig. 1e). Henceforth, the colour combinations of the chromatic flicker stimuli are encoded COLOUR1/COLOUR2, in which COLOUR1 and COLOUR2 refer to the single letter representation of the colour in each phase, i.e. A/R indicates flicker between amber and red.

## Methods

The study was approved by the institutional review board at the Technical University of Denmark’s Department of Applied Mathematics and Computer science (COMP-IRB-2021-01), and the data was collected in accordance with the Declaration of Helsinki. The analysis was conducted according to a pre-specified statistical analysis plan. The data set is available from the corresponding author on reasonable request.

### Experimental setup

Thirty healthy subjects (15 females) with a mean age of 28 ($\pm 8$) years were recruited to participate in the study. They received written information about the study at least 24 hours in advance and verbal information on the day of the study before signing informed consent for their inclusion.

A standalone six-LED device (Light Therapy System, OptoCeutics ApS, Denmark) was used to provide chromatic flicker stimulation at 40 Hz^50^. The six LEDs had spectral profiles centred around blue, cyan, green, lime, amber, and red, respectively, and allowed for generation of 15 dyadic heterochromatic combinations. Following the procedure in Ref.^50^, each LED pair was luminance-matched to minimise the perceived flicker, and the 15 heterochromatic combinations were matched to the same illuminance, ensuring exactly the same visual response (see Sect. S.1 of the supplementary material).

**Figure 2:**
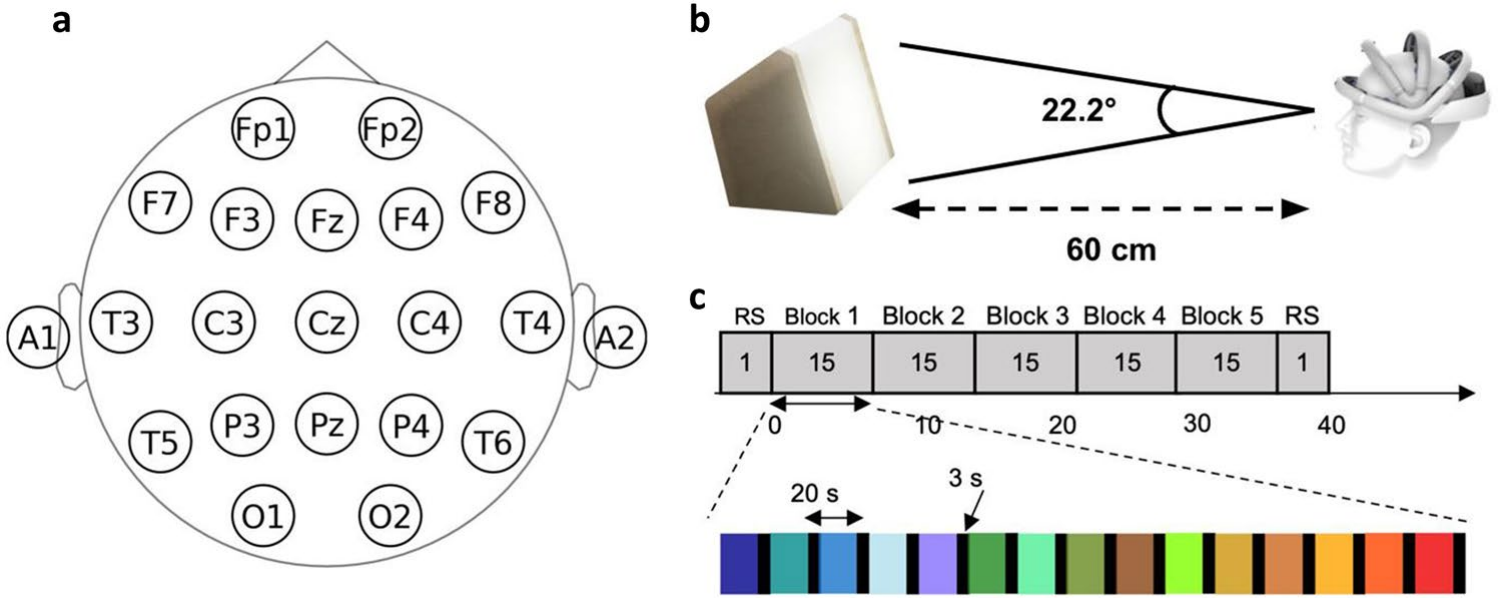
Experimental Overview: (**a**) The International 10-20 system for electrode placement for the ZETO system. (**b**) A programmable visual stimulation system is situated 60 cm from the subject covering 22.2 degrees of the horisontal field of view. (**c**) The statistical design is a within-subject repeated measurement design with one epoch per time block (B1, B2, B3, B4, B5) per stimuli. Note that RS denotes the baseline (resting state). Stimuli are balanced and randomised within blocks throughout the experiment.

Subjects were presented with repeated trials of stimulation with each of the LED configurations in a single session. During stimulation, EEG was recorded using the EEG Monitoring Device by Zeto Inc. with 19 channels configured according to the 10-20 system (Fig. 2a) and a sampling rate of 500 Hz and linked mastoid reference. The subject was placed 60 cm from the stimulation device and requested to sit in a relaxed position with their gaze fixed on the centre of the device (Fig. 2b).

The session was split into five blocks, and every LED combination appeared once within each block. Trials had a duration of 20 seconds, separated by a 3 second interval with no stimulation (Fig. 2c). The order of the light stimuli within the block was randomised during data collection to eliminate any order effect. This resulted in a total of 2250 recorded trials across the 30 subjects.

Prior to and after the stimulation paradigm, 120-seconds baselines with eyes open (60 s) and eyes closed (60 s) were also recorded. The total EEG recording duration was around 34 minutes, excluding instructions and mounting of the electrodes. In total, the experiment took no longer than 45 minutes.

### EEG data preprocessing and analysis

The EEG data was cleaned and processed in Python using tools from the MNE library^52^. Line noise in the recordings was reduced using a finite impulse response (FIR) notch filter with a bandwidth of 0.5 Hz and transition bandwidth of 0.5 Hz. Signals were subsequently band-pass filtered from 1 Hz to 50 Hz using a FIR filter with transition bandwidths of 1 Hz and 12.5 Hz for the lower and higher cut-offs.

Data was manually inspected and segments marked as bad at an indication of sufficiently elevated amplitude. Such segments were typically instances of subject movement or excessive electromyographic contamination from tense subjects. Individual channels with persistently noisy data throughout a session were marked as bad. Independent component analysis (ICA) was applied to remove artefact influences such as blinking. After this step, the bad channels were interpolated using spherical splines. Epochs of 20 seconds, corresponding to individual trials, were extracted from the EEG using the annotations marked for onset of stimulus. All epochs containing bad data segments were rejected prior to further analysis. In total, 700 epochs were marked as bad, reducing the final number of total epochs from 2250 to 1550.

The SSVEP response to each epoch was quantified as the signal-to-noise ratio (SNR) at 40 Hz, defined as the ratio of the peak power at 40 Hz compared to the neighbouring frequencies as in Ref.^53^.

The power spectral density (PSD) was estimated using the Welch method with a 9 s Hann window function with 50% overlap. The PSD is used for both signal and noise power estimation. The signal power is defined at 40 Hz in the PSD, and the noise power is the average power over the neighbouring 38-39 Hz and 41-42 Hz bands. The final reported SNR is obtained by averaging over all channels and converted to units of dB for use as the response variable in statistical modeling. See Eqs. (S.1) to (S.10) in Section S.2 of the supplementary material for details regarding SNR estimation.

### Design and analysis of experiment

The statistical analysis investigated the relation between stimuli and SSVEP response (SNR [dB]) by using mixed effects models. Subject, stimulus, blocks, and all interactions were included as factors in the statistical design of the experiment. While these were initially balanced, the rejection of epochs in pre-processing resulted in imbalance across all experimental factors. The stimuli were treated as fixed effects, while the subjects and blocks were treated as random effects to allow for inference about the stimuli beyond the sampled subjects and blocks.

The response was box cox-transformed in order not to violate model assumptions. The random effect structure was reduced using likelihood ratio tests, testing the null hypothesis $H_0: \sigma _{r}^2 = 0$ against the alternative $H_A: \sigma _{r}^2 > 0$, where $\sigma _{r}^2$ is a given random effect variance. As this is a test on the boundary of the parameter domain, the p-value was adjusted by $\frac{1}{2}$. Both the interaction effect between stimulus and block and the block main effect were non-significant ($\alpha =5\%; p_{stimulus:block} = 0.5000, p_{block} = 0.1181$). However, only the block-stimulus interaction was removed, as the block is part of higher order significant random effect and intrinsic for the experimental design. The fixed effect structure was reduced using F-tests with Satterthwaite’s denominator degrees of freedom. The stimulus effect was significant with a p-value less than 0.0001. The final model is given by Eq. (1), in which ${\text {SNR}}^{0.48}_{ijk}$ refers to the box-cox transformed SNR response (with $\lambda = 0.48$, see Eq. (S.12) in the supplementary material) to the epoch with stimulus *i*, subject *j*, and block *k*:

1 $$\begin{aligned} \begin{aligned} {\text {SNR}}^{0.48}_{ijk} = \mu&+ \alpha _i + a_j +b_k +c_{jk} +f_{ij} +\varepsilon _{ijk}, \\ \text{ where }&\\&i = 1, 2, \ldots , 15, \quad j = 1, 2, \ldots , 30, \quad k = 1, 2, \ldots , 5, \\&a_j \sim \mathscr {N}\left( 0, \sigma _a^2\right) , \\&b_k \sim \mathscr {N}\left( 0, \sigma _b^2\right) , \\&c_{jk} \sim \mathscr {N}\left( 0, \sigma _c^2\right) , \\&f_{ij} \sim \mathscr {N}\left( 0, \sigma _f^2\right) , \\&\varepsilon _{ijk} \sim \mathscr {N}\left( 0, \sigma ^2\right) . \end{aligned} \end{aligned}$$

and $\alpha$, *a*, and *b* are the stimulus, subject, and block main effects, respectively, while *c* and *f* are the subject:block and subject:stimulus interaction effects, respectively.

The principal post-hoc analysis compares the stimuli pairwise. With 15 stimuli, there are 105 pairwise comparisons. Thus, all p-values of the pairwise comparisons are corrected for multiple comparisons by Tukey’s method, comparing a family of 15 estimates.

Details about the statistical modelling are presented in section S.3 of the supplementary material.

## Results

### Estimated mean SSVEP response

**Figure 3:**
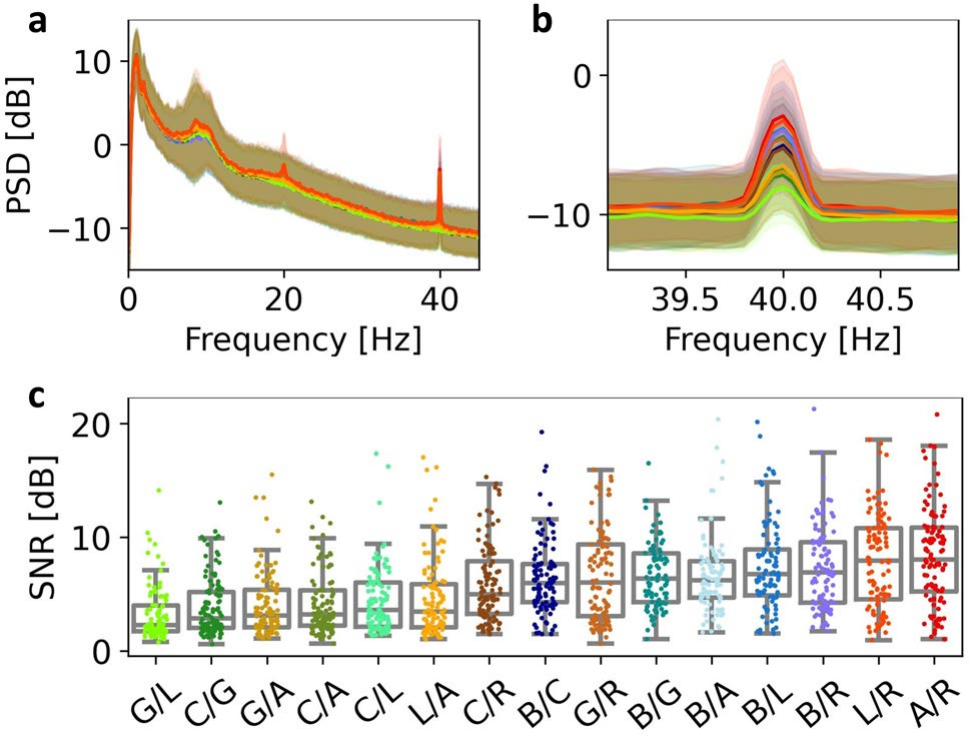
PSD and SNR by Stimulus: The SSVEP responses to stimuli are visible from the power spectra and are quantified by the 40 Hz signal-to-noise ratio (SNR) in each epoch. Each colour in the plots correspond to a chromatic flicker stimulus and is given by the *x*-axis of panel b, encoded COLOUR1/COLOUR2, in which COLOUR1 and COLOUR2 refer to the single letter representation of the colour in each phase - i.e. A/R indicates flicker between amber and red. (**a**,**b**) Power spectral density (PSD) of the SSVEPs. For each stimulus, a full-bodied line indicates the grand averages of epochs across subjects and channels, while shaded areas indicate $\pm 1$ standard deviation. (**c**) Boxplots of 40 Hz SSVEP SNR responses averaged across channels, grouped by stimulus, and sorted by mean SNR. One point indicates one epoch.

**Figure 4:**
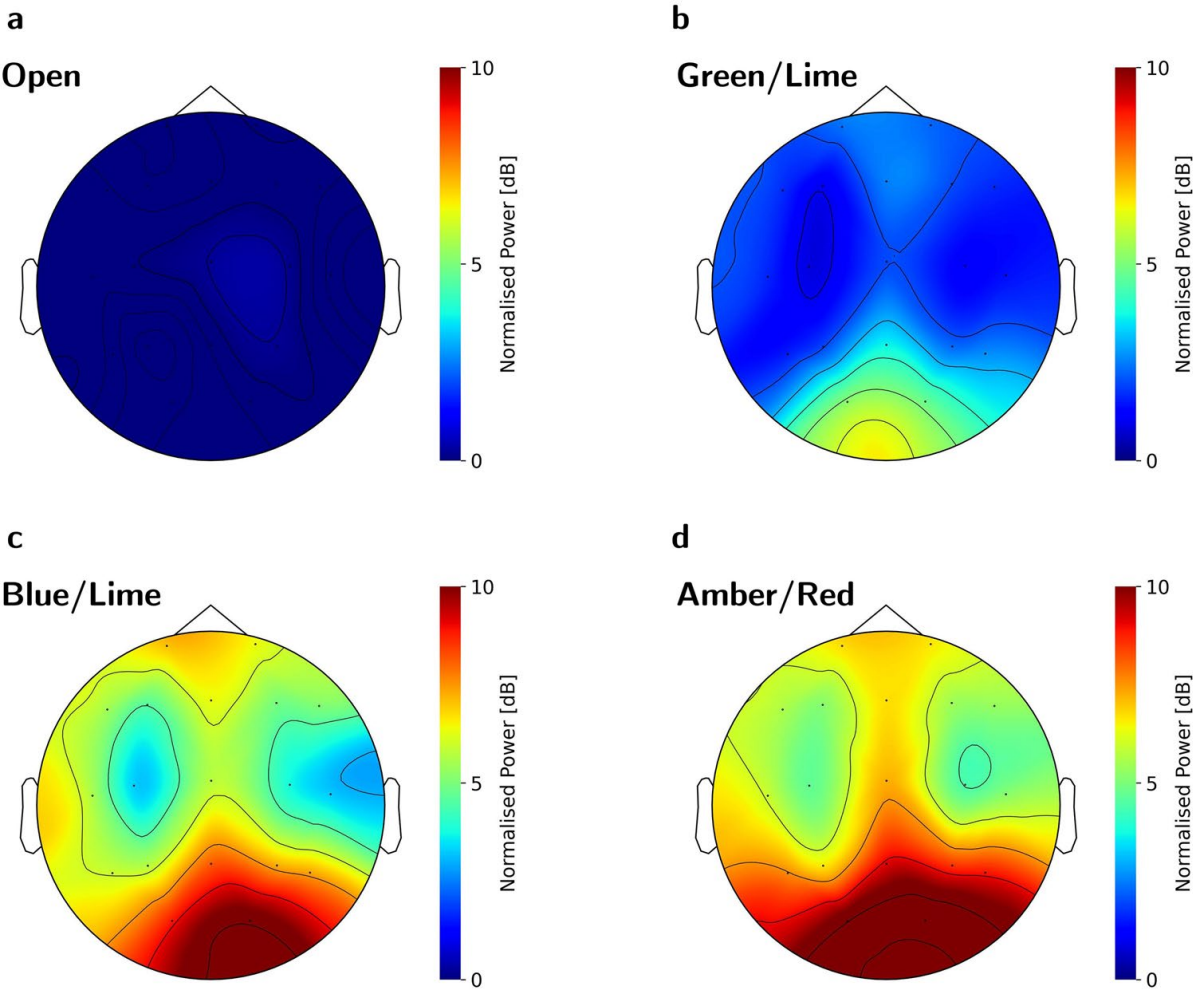
Topographic Maps of 40 Hz Signal: The 40 Hz power of the EEG normalised to the average [38;39] Hz and [41;42] Hz power is shown across the 19 EEG channels as topographic maps for four representative conditions (see Figs. S.9 to S.13 in section S.4 of the supplementary material for all topographic maps and by-channel relative 40 Hz power estimates). (**a**) Baseline condition with no stimulation and open eyes results in no increase of 40 Hz power. (**b**) The Green/Lime stimulus condition (which has the lowest global response) increases the 40 Hz power predominantly at channels near the occipital lobe, though some frontal propagation is evident. (**c**,**d**) Stimulus conditions with either Blue or Red such as Blue/Lime (**c**) and Amber/Red (**d**) conditions increase the 40 Hz power the most, and the signal is registered at electrodes across the entire scalp, though the power increase is highest at the electrodes near the occipital lobe. All plots are created from grand averages across repetitions and subjects.

**Figure 5:**
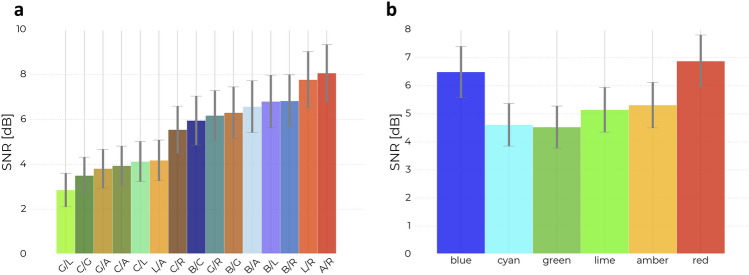
Estimated Mean Steady-State Visually Evoked Potential: Estimated marginal means (EMMs) of the SSVEP responses are plotted along with error-bars their 95% confidence intervals. Estimates are given both for colour combinations and for single colour contributions. (**a**) EMMs of the 15 stimuli of heterochromatic flicker combinations in the BCGLAR space. Combinations with red or blue are found to have a higher response than combinations without. The largest span between two consecutive values are found between L/A and C/R, which marks the boundary for combinations with and without red or blue. For an overview of the significantly different pairs, see the compact letter display (CLD) column in Table 1. (**b**) EMMs of the responses grouped by the 6 colours that the heterochromatic stimuli are composed of. If for example, the stimulus is A/R, it contributes to both the amber and the red group. This emphasises that stimuli containing either red and/or blue evoke a higher SSVEP response.

From the PSD estimates presented in Fig. 3a and b, it is evident that all 15 stimuli evoked a 40 Hz SSVEP response, though the magnitude of the 40 Hz SNR varied. Topographical maps of the relative 40 Hz power across EEG channels are plotted for four representative conditions in Fig. 4. These indicate that the stimuli increase the 40 Hz power predominantly at electrodes near the occipital lobe, though an increase is seen in electrodes across the scalp in even the stimulus condition with the lowest response (Green/Lime) compared to the baseline condition without stimulation (Fig. 4a,b). The occipital and global increases are especially clear for stimulus conditions containing Blue and/or Red (see Fig. 4c,d for representative high response topographic maps and Figs. S.9–S.13 in Section S.4 of the supplementary material for all topographic maps and by-channel relative 40 Hz power estimates). The estimated marginal mean (EMM) SSVEP responses for the stimuli are presented in order of EMM size along with their 95% confidence intervals in Table 1 and Fig. 5a and contrast significance indicated by compact letter display (CLD). A pairwise P-value plot of all stimulus contrasts is also found in Fig. S.8 of the supplementary material. The response was on average the highest for the A/R combination and lowest for the G/L combination.

**Table 1:** Sorted Estimated Marginal Mean SSVEP: Estimates are presented for each stimulus along with a 95% confidence interval.

Stimulus	Estimates	95% CI	CLD
Amber/Red (A/R)	8.06	6.79, 9.34	e
Lime/Red (L/R)	7.77	6.51, 9.03	de
Blue/Red (B/R)	6.82	5.65, 8.00	cde
Blue/Lime (B/L)	6.80	5.63, 7.97	cde
Blue/Amber (B/A)	6.57	5.42, 7.73	cde
Blue/Green (B/G)	6.30	5.15, 7.45	cde
Green/Red (G/R)	6.18	5.06, 7.29	cde
Blue/Cyan (B/C)	5.95	4.86, 7.04	cd
Cyan/Red (C/R)	5.54	4.50, 6.59	bc
Lime/Amber (L/A)	4.18	3.27, 5.08	ab
Cyan/Lime (C/L)	4.13	3.23, 5.02	ab
Cyan/Amber (C/A)	3.93	3.06, 4.81	ab
Green/Amber (G/A)	3.81	2.94, 4.67	a
Cyan/Green (C/G)	3.50	2.67, 4.32	a
Green/Lime (G/L)	2.85	2.11, 3.60	a

The estimates are back-transformed to the original SSVEP domain. The last column shows a compact letter display (CLD) of the pairwise comparisons. If two stimuli share the same letter, they are not statistically different in terms of SSVEP (pairwise comparison has a p-value above 5%), contrary if they do not share the same letter, then they are statistically different in terms of SSVEP (pairwise comparison has a p-value below 5%). A dashed line marks the boundary between stimuli that contain red or blue and those that do not.

From the ordered EMMs, there is an apparent boundary between responses to colour combinations that contain red or blue and those that do not. Notice that the largest span between two consecutive values is between C/R and L/A, which marks this boundary (indicated in Table 1 by a dashed line).

Overall, the study found that no consecutive stimuli in the ordered EMMs are pairwise significantly different. Stimuli at the top of the table are, in general, not significantly different from each other, and stimuli at the bottom of the table are, in general, not significantly different from each other. However, stimuli from the top of the table are, in general, significantly different from stimuli at the bottom of the table. All stimulus comparisons are presented as a paiwise p-value plot in Fig. S.8 in section S.3 of the supplementary material.

### Red and blue increase SSVEP

There is a strong indication from the EMMs for a higher SSVEP response from stimuli that contain red and/or blue. Figure 5b presents EMMs and 95% confidence intervals of responses grouped by single colours, such that a colour combination of e.g. red and blue contributes to both of these individual colours. A t-test comparing the combinations with red and/or blue to those without (E = 2.93, T = 14.042, df = 84.2, p < 0.0001) indicates a significant difference after correcting for multiple comparisons. A more conservative Bonferroni-corrected significance level of $\alpha = 0.05/106 = 0.00047$ was used, as this test was not pre-specified (the test was number 106 after 105 pairwise comparisons of the 15 stimuli). This indicates that stimuli that contain blue and/or red colours evoke a significantly larger SSVEP than stimuli that do not contain these colours.

### SSVEP response variability

As evident from Fig. 3c, SSVEP response variance (prior to box-cox transformation) in general increased with the mean response size, which also affects the confidence intervals for the estimated means seen in Fig. 5a. The largest sources of variance were related to inter-subject variability ($\hat{\sigma }_a^2=0.866^2$) and the subject-stimulus interaction ($\hat{\sigma }_f^2=0.703^2$). The subject-stimulus interaction suggests some degree of preference towards certain stimuli between subjects. The block effect and the block-subject interaction introduced less variability with estimated variances of $\hat{\sigma }_b^2=0.056^2$ and $\hat{\sigma }_c^2=0.187^2$, respectively.

## Discussion

The brain response to different colours of light is important since it impacts the brain’s alertness, brain waves, vision and circadian system. The impact of blue and green light on human emotional state has been studied by examining brain responses^54^ and the impact on the human circadian clock^55–57^. Steady-state visually evoked potentials from visual stimulation based on red, blue and green LEDs are also studied for optimal use in BCI-systems^43–48^. The brain activity arising from white light flickering at 40 Hz has been investigated as a potential treatment for Alzheimer’s^23–29^. However, in contrast to the above investigations, our work presents the brain’s response to 40 Hz stimulation using different colours of light in a heterochromatic flicker configuration.

This study demonstrated that stimulation with 40 Hz heterochromatic flicker using all 15 dyadic combinations of monochromatic colours from the BCGLAR colour space can entrain a 40 Hz SSVEP response in healthy volunteers. The size of the response was dependent on the choice of colours and the subject, and there was a degree of subject-dependent preference for the colour combinations. Inference to the wider population must, however, take into account that the vast majority of subjects were 20-30 years old, though there was one subject in each of the 30-40, 40-50, and 60-70 years ranges.

The magnitude of the response seems to be dependent on whether or not red and/or blue were among the chosen colours in the stimuli. This suggests the presence of “high SSVEP zones” in both ends of the visual spectrum, while the SSVEP responses to colour combinations in the middle of the spectrum are relatively depressed. An explanation for the higher response to red and blue may be the higher radiant flux of these compared to the mid-spectrum colours, potentially detected by ipRGCs as an increase in non-image forming irradiance information^34^, though other currently unknown mechanisms may also be involved. Since the colours where matched by luminous flux (i.e. the perceived ’light intensity’), and the human sensitivity colour varies greatly, a higher radiant flux can be delivered in the extremes of the band of visible light compared to the middle. Matching luminance rather than irradiance is important for use of chromatic flicker in human light therapy, as luminance imbalance introduces luminance flicker, and the perception of flicker may affect the user experience.

Colour-opponency may provide an explanation for why the highest SSVEP was achieved by the combination of spectrally similar red and amber colours (A/R). Though the absorbance spectra of the S-, M-, and L-cones overlap, the strongest activation of the R/G axis is through absorption of a long wavelength red light by the L-cones (see the colour-opponency axes in Fig. 1b). For the B/Y axis, the strongest activation is through either S-cone absorption of short wavelength blue light or yellow light, which is achieved through a mixture of red and green light. As such it is possible that the largest SSVEP response measured downstream from the RGCs would come from maximising the activation of either L- or S-cones. This presents a possible explanation for the high SSVEP response to the A/R combination. The red phase activates the L-cone substantially while mostly bypassing the M-cone. Simultaneously, the amber phase activates the B/Y pathway by its absorbance of yellow.

This study also confirms previous findings of a high SSVEP SNR achieved by R/G heterochromatic flicker^43,45^. More importantly though, the study demonstrated that it is possible to evoke SSVEPs using spectrally similar colours achieved by combining colours of the BGGLAR space. There is even a prospect that spectrally similar colours such as the A/R combination can evoke greater response than R/G, as is the case here, though the difference between the two was not significant in this study. These findings highlight the complex nature of generating SSVEPs.

While our findings add nuance to the field of brain stimulation through the visual pathway, its implications for BCI systems are less clear. The aim of BCI systems is typically to discern user intentions (such as identifying strokes of a keyboard), and as this requires the display of information on a screen (such as an on-screen keyboard), it limits the systems to display the colours available from monitors that usually rely on RGB combinations. As such, the experiments conducted in other studies focused on RGB-based BCI systems are not all readily reproducible with the setup used in this study^43–48^. This limitation arises primarily from the BCGLAR stimulation device being based on diffusion of a few LED point sources into a homogeneous screen that lacks spatial differentiation of colour achieved by an array of sources, and thus it can not display information as a monitor can.

Evoking brain oscillations using heterochromatic flicker of spectrally similar light sources might, however, prove beneficial in the domain of brain stimulation as it is possible to minimise potential side effects associated with exposure to the flicker of spectrally different colours. This warrants future experiments into the comfort associated with stimuli used in this study as well a comparison to other the stimuli currently used in clinical studies of AD such as stroboscopic flicker^58^ and invisible spectral flicker^49^.

While there was an overall higher response from stimuli that contained red and/or blue, the subject-stimulus interaction also introduced significant variability. This indicates a degree of subject-specific preference towards certain stimuli. Given that the inter-subject variability was the largest source of variance, it is natural to consider that this variability also affects the impact from individual stimuli differently. It may therefore be useful to pursue a personally optimised 40 Hz chromatic flicker stimulation paradigm with colour combinations selected for the individual based on a trade-off between perceived flicker and SSVEP magnitude.

All the above suggestions of alternatives to 40 Hz stroboscopic flicker stimulation are made under certain assumptions. First, the responses of the young healthy population recruited for this study are not guaranteed to generalised to an older or even a pathological population.

Then, the assumption is that the preliminary clinical efficacy for therapy of AD previously shown in mouse models and human trials is proportional to the size of the acutely measured 40 Hz SSVEP response. Such an assumption is associated with a high degree of uncertainty, as the exact mechanism of action remains unclear and could be specifically related to the spectral composition of the stroboscopic flicker used in^23–29^, and which^49^ also matched with their visual stimulation.

However,^23^ show at least some degree of specificity with regard to the stimulation paradigm, showing that stroboscopic flicker at 20 Hz and 80 Hz does not results in the same neuroprotective effects as 40 Hz in mouse models of AD. The specificity may suggest that the benefits are more associated with the choice of frequency than the medium of stimulation. Supporting this idea are clinical findings such as those of^59^ that used 40 Hz repetitive transcranial magnetic stimulation over the bilateral angular gyrus in patients with probable Alzheimer’s disease, resulting in significantly improved cognitive function, prevention of gray matter volume loss, and improved functional connectivity. Furthermore,^30,60,61^ combined 40 Hz stroboscopic flicker with 40 Hz modulated clicking sounds in clinical trials on the safety, feasibility, and efficacy of treatment in the AD population and found improvements on functional abilities and preliminary indications of immune and network effects, respectively.

If the above can be considered indications that the pathway for stimulation is of less importance than the choice of 40 Hz frequency, there is reason to pursue chromatic flicker as an alternative stimulation paradigm.

As the research of 40 Hz visual brain stimulation for therapy of AD transitions to its clinical phase, it may be advantageous to use an alternative to stroboscopic stimulation as its inherent flicker makes it ill-suited for human use due to its CFF which is at least 15 Hz higher than the stimulation frequency.

This study demonstrated that heterochromatic flicker with spectrally similar dyadic phases could be an alternative for evoking 40 Hz SSVEPs. Specifically, the inclusion of at least one colour from either extreme of the visual spectrum (blue and/or red) is more important than using two colours that are spectrally far apart. In this sense, the observed zones of high SSVEP responses at the colour extremes may be explained by maximal activation of either of the two colour opponency axes through use of red and/or blue. This effect allows for the pairing of red or blue with spectrally similar colours such as amber or cyan, which may fuse more easily the spectrally dissimilar colours while retaining a strong SSVEP response. An alternative or complementary explanation for the results are the differences in cone sensitivity colours across the spectrum, leading to a higher radiant flux of blue and red colours when matching based on luminous flux, and thus, perhaps, a stronger activation of the ipRGCs.

Thus, for human applications of 40 Hz visual stimulation such as AD, efficacy of therapy may be improved by administering heterochromatic flicker with one phase of blue or red and another phase of a spectrally similar colour. By reducing the flicker associated discomfort, the adherence to therapy is expected to rise, perhaps increasing overall treatment effectiveness. The choice of colour combinations may even be tailored to the individual to optimise the trade-off between perceived flicker and evoked response.

While the presented findings are valid for the wider population of young healthy controls, they are not guaranteed to generalise to the older- or even AD-pathological populations. Similarly, all recommendations are made under the assumption that the presence and/or magnitude of the SSVEP is in some way proportional to the medical benefits found in previous pre-clinical and clinical studies of 40 Hz stimulation for AD.

### Supplementary Information

Supplementary Information.
