# Supplementary material for: Brain stimulation with 40 Hz heterochromatic flicker extended beyond red, green, and blue
Source: Sci Rep. 2024 Jan 25;14:2147. doi: 10.1038/s41598-024-52679-z (PMC10810780; doi:10.1038/s41598-024-52679-z)
Supplement: Supplementary file 1 — Supplementary Information. [file 41598_2024_52679_MOESM1_ESM.pdf]

Supplementary Material

Brain Stimulation With 40 Hz Heterochromatic Flicker Extended Beyond Red, Green, and Blue

S.1 Light Specifications

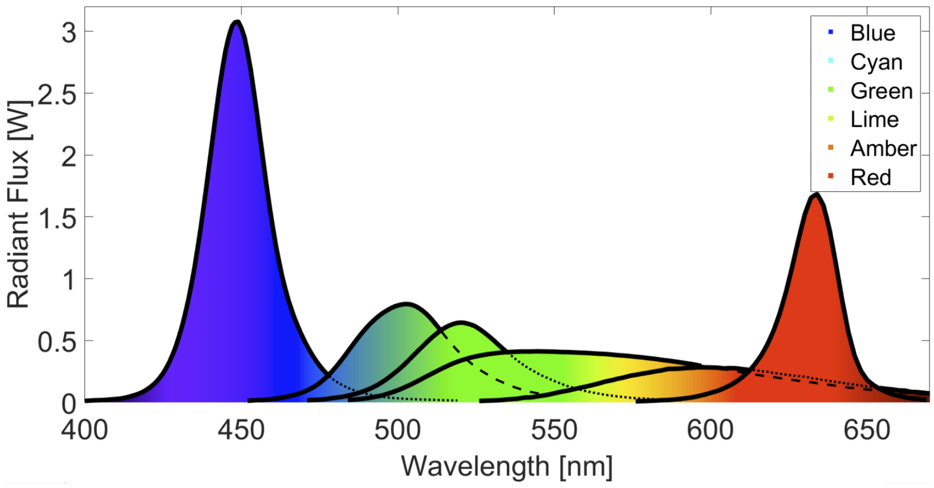

Figure S.1. Spectral distributions of each of the six LEDs: The spectrum of each LED is outlined by a full-bodied black line, except where spectra overlap, in which case the LED of lower centre wavelength continues as a dotted or dashed line (alternating). Evidently, the blue and red LEDs have much narrower spectra than the central LEDs, and the lime and amber LEDs are especially wide-band.

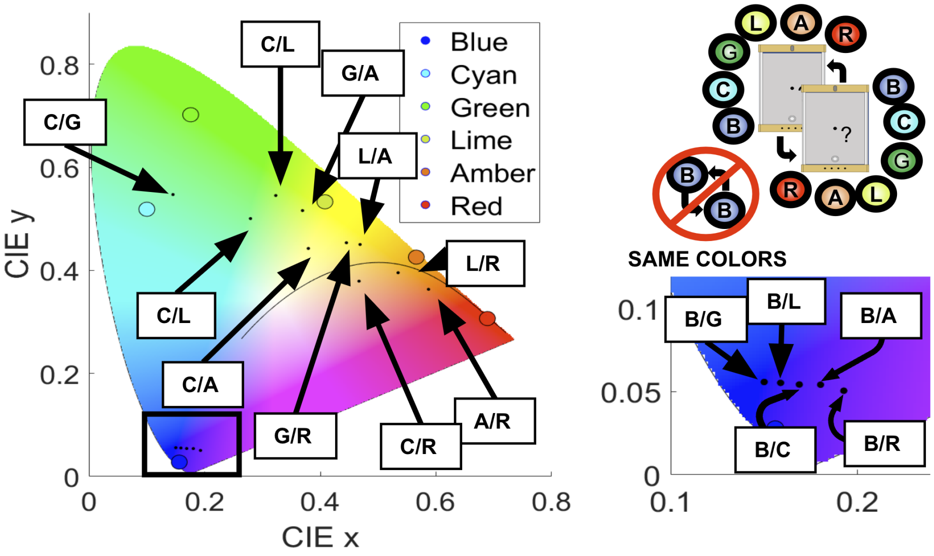

Figure S.2. CIE 1931 chromaticity coordinates: The chromaticity is plotted for the individual LEDs and heterochromatic LED combinations. Combinations containing blue are emphasised in the inset for clearer distinction.

Table S.1. CIE 1931 chromaticity coordinates: Coordinates of each of the resulting spectra from the heterochromatic LED combinations.

Color Combination		Abbreviation	CIE1931xy
Blue	Cyan	B/C	{0.1556 ; 0.0559}
Blue	Green	B/G	{0.1588 ; 0.0553}
Blue	Lime	B/L	{0.1688 ; 0.0543}
Blue	Amber	B/A	{0.1802 ; 0.0542}
Blue	Red	B/R	{0.1927 ; 0.0506}
Cyan	Green	C/G	{0.1451 ; 0.5471}
Cyan	Lime	C/L	{0.2791 ; 0.5005}
Cyan	Amber	C/A	{0.3793 ; 0.4425}
Cyan	Red	C/R	{0.4670 ; 0.3790}
Green	Lime	G/L	{0.3229 ; 0.5452}
Green	Amber	G/A	{0.3692 ; 0.5159}
Green	Red	G/R	{0.4452 ; 0,4535}
Lime	Amber	L/A	{0.4688 ; 0.4501}
Lime	Red	L/R	{0.5346 ; 0.3954}
Amber	Red	A/R	{0.5872 ; 0.3631}

S.2 EEG Processing

EEG data was cleaned and processed in Python version 3.9.7. Both pre-processing and PSD estimation by the Welch method (eqs. (S.1) to (S.6)) was performed using tools from version 0.24.1 of the MNE library. The SNR value of an epoch was calculated according to the following procedure:

For each of M sub-samples with $R = M/2 = 50\%$ overlap, the m'th sub-sample with length L is defined as

$$x_{ikm}(n) = x_k((mRL + n) \quad n = 0, 1, \ldots, L-1 \quad m = 0, 1, \ldots, M-1, \tag{S.1}$$

where $L = N/M$ is the length of each segment, and $x_{ik}(n)$ is the original one-dimensional time series of a channel, k, in epoch i with length N. This is weighted by the Hann window

$$w(n) = 0.5\left(1 - \cos\left(\frac{2\pi n}{L+1}\right)\right)\text{boxcar}_L(n) \quad n = 0, 1, \ldots, L-1, \tag{S.2}$$

where

$$\text{boxcar}_L(n) = \begin{cases} 1 & 0 \leq n \leq L-1 \\ 0 & \text{Otherwise} \end{cases}. \tag{S.3}$$

Thus, the sub-sample tapered by a Hann filter is expressed as

$$x^w_{ikm}(n) = w(n) \odot x_{ikm}(n), \tag{S.4}$$

where $\odot$ is the elementwise (Hadamard) product. From this, the Welch PSD for epoch i is obtained as the average over the periodograms of the tapered sub-samples:

$$\hat{S}^w_{ik}(f) = \frac{1}{M}\sum_{m=M}\text{PSD}_{ikm}(f), \tag{S.5}$$

in which

$$\text{PSD}_{ikm}(f) = \frac{1}{L}\left|\sum_{n=1}^{L} x^w_{ikm}(n)e^{-j2\pi fn}\right|^2, \quad f = -F, -F + \frac{f_s}{L}, \ldots, F \tag{S.6}$$

for sampling frequency f_s and $F = \frac{f_s}{2}$. From this and with stimulation frequency $f_i = 40$ Hz, the SNR is quantified (see fig. S.3) as:

$$\text{SNR}_{ik}(f_i) = \left(\frac{\hat{S}^w_{ik}(f_i)}{\text{Noise}_{ik}(f_i)}, \right) \tag{S.7}$$

in which the noise is average power over the neighbouring bands defined by

$$\text{Noise}_{ik}(f_i) = \frac{\sum_f \hat{S}^w_{ik}(f) \odot k(f, f_i)}{\sum_f k(f, f_i)}, \tag{S.8}$$

where $\odot$ is the Hadamard product, and the kernel function, $k(f, f_i)$, selects the bands neighbouing f_i by

$$k(f, f_i) = \begin{cases} 1 & \text{for } f - 2\text{Hz} < f_i < f - 1\text{Hz and } f + 1\text{Hz} < f_i < f + 2\text{Hz} \\ 0 & \text{Otherwise} \end{cases}. \tag{S.9}$$

Within each epoch, the 40 Hz SNR values, $\text{SNR}_{ik}(40\text{ Hz})$, are averaged over $K = 19$ channels to obtain the mean 40 Hz SNR of i and converted to dB:

$$\text{SNR}_i(40\text{ Hz}) = 10\log_{10}\left(\frac{1}{K} \sum_{k=0}^{K-1} \text{SNR}_{ik}(40\text{ Hz}) \right). \tag{S.10}$$

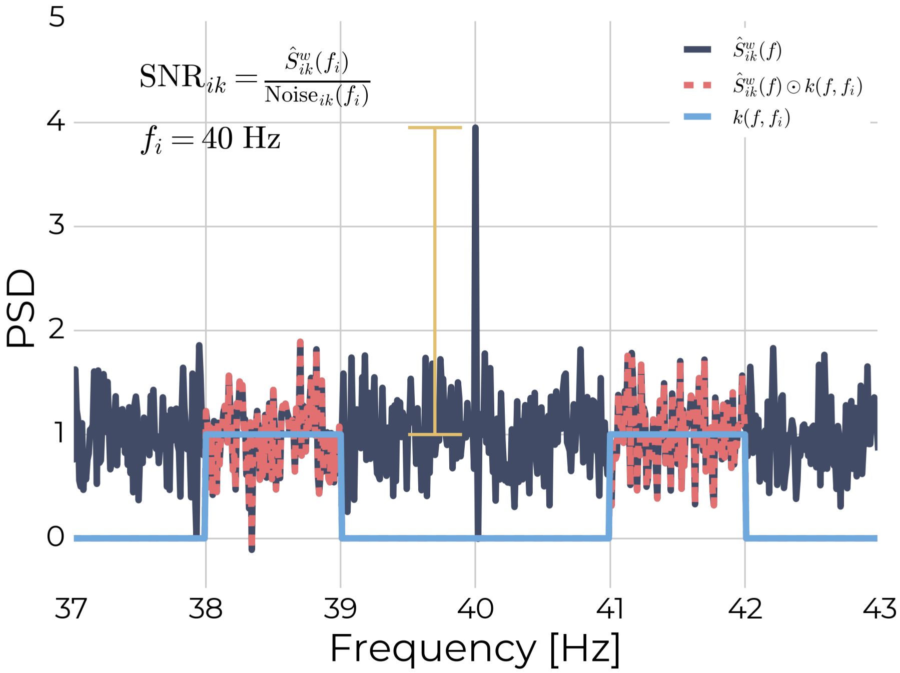

Figure S.3. Signal-to-Noise Ratio Estimation: The Signal-to-Noise Ratio (SNR) is quantified as the $f_i = 40$ Hz power normalised by the mean power of the [38;39] Hz and [41;42] Hz bands (eq. (S.7)). Here, $\hat{S}^w_{ik}(f)$ is the power spectral density of trial i and channel k, $k(f, f_i)$ is the kernel function for selecting the noise bands described by eq. (S.9), and $\text{Noise}_{ik}(f_i)$ is described by eq. (S.8).

S.3 Statistical Analysis

The statistical analysis was conducted using R (version 4.1.2). The mixed effects model library lme4 library (version 1.1.27.1) was used to estimate the mixed models, the lmerTest library (version 3.1.3) was used for significance tests, and the emmeans library (version 1.8.3) was used in the post-hoc analysis.

The initial linear mixed effects model of the experiment is described by eq. (S.11), in which all terms are present prior to model reduction. Here, $\mathrm{SNR}_{ijk}^{0.48}$ refers to the box-cox transformed SNR response to the epoch with stimulus i, subject j, and block k:

$$\begin{aligned} &\mathrm{SNR}_{ijk}^{0.48} = \mu + \alpha_i + a_j + b_k + c_{jk} + d_{ik} + f_{ij} + \varepsilon_{ijk}, \\ &\quad \text{where} \\ &\qquad i = 1,2,\ldots,15, \quad j = 1,2,\ldots,30, \quad k = 1,2,\ldots,5, \\ &\qquad a_j \sim \mathcal{N}\left(0,\sigma_a^2\right), \\ &\qquad b_k \sim \mathcal{N}\left(0,\sigma_b^2\right), \\ &\qquad c_{jk} \sim \mathcal{N}\left(0,\sigma_c^2\right), \\ &\qquad d_{ik} \sim \mathcal{N}\left(0,\sigma_d^2\right), \\ &\qquad f_{ij} \sim \mathcal{N}\left(0,\sigma_f^2\right), \\ &\qquad \varepsilon_{ijk} \sim \mathcal{N}\left(0,\sigma^2\right). \end{aligned} \tag{S.11}$$

and α, a, and b are the stimulus, subject, and block main effects, respectively, while c, d, and f are the subject:block, stimulus:block, and stimulus:subject interaction effects, respectively.

As shown in fig. S.4a, the SSVEP response was not normally distributed, but rather skewed right, and the fitted versus residual values presented in fig. S.4b shows an increase in variance with the mean. Thus the responses needed to be transformed to meet the model assumptions of eq. (S.11).

The applied box-cox transformation is expressed by eq. (S.12) for response y_i to epoch i:

$$y_i^{(\lambda)} = \begin{cases} \frac{y_i^\lambda - 1}{\lambda} & \lambda \neq 0 \\ \log\left(y_i\right) & \lambda = 0 \end{cases}, \tag{S.12}$$

and is optimised for λ by maximisation of the log-likelihood of the transformed data. For simplicity, the likelihood is based on a fixed effect model corresponding to the mixed model in eq. (S.11). This should not make a huge difference for the optimal value of λ. The function `boxcox` from the R library `MASS` is used to optimise the likelihood over λ. The optimised value is found at $\lambda = 0.48$ with an approximate 95% confidence interval of $[0.42, 0.54]$. The suggested box-cox transformation is thus close to a square root transformation of the response (0.5 is within the approximate 95% CI). The transformed distribution is presented in fig. S.5a, and the fitted vs residual values for the model of the transformed data is presented in fig. S.5b. In fig. S.6, the residuals against all effects in the model are plotted, and qqplots for the residuals and random effects are presented in fig. S.7.

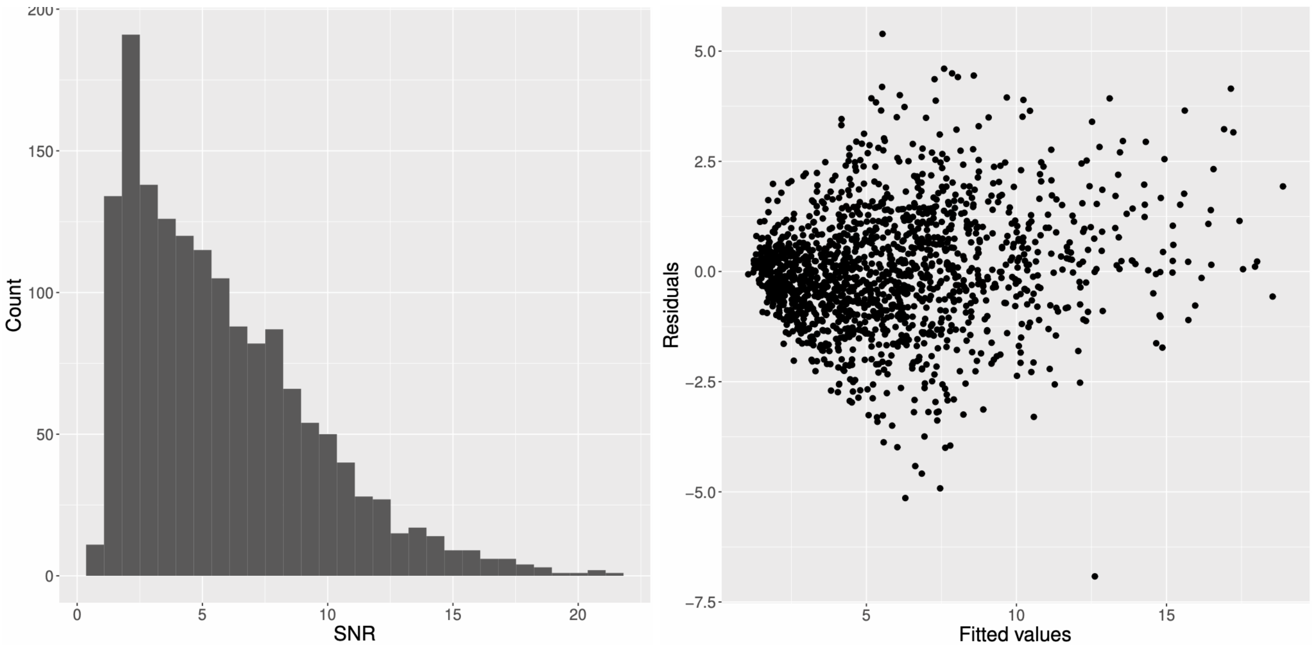

(a) Original Response.

(b) Fitted vs. Residual Values.

Figure S.4. Initial Model Distribution and Fit: a) In the original SSVEP space, the response was not normally distributed, but rather skewed right. Thus they had to be transformed into normal distribution to use the mixed effect linear model. **b)** Using the box-cox transformation, the response was normalised.

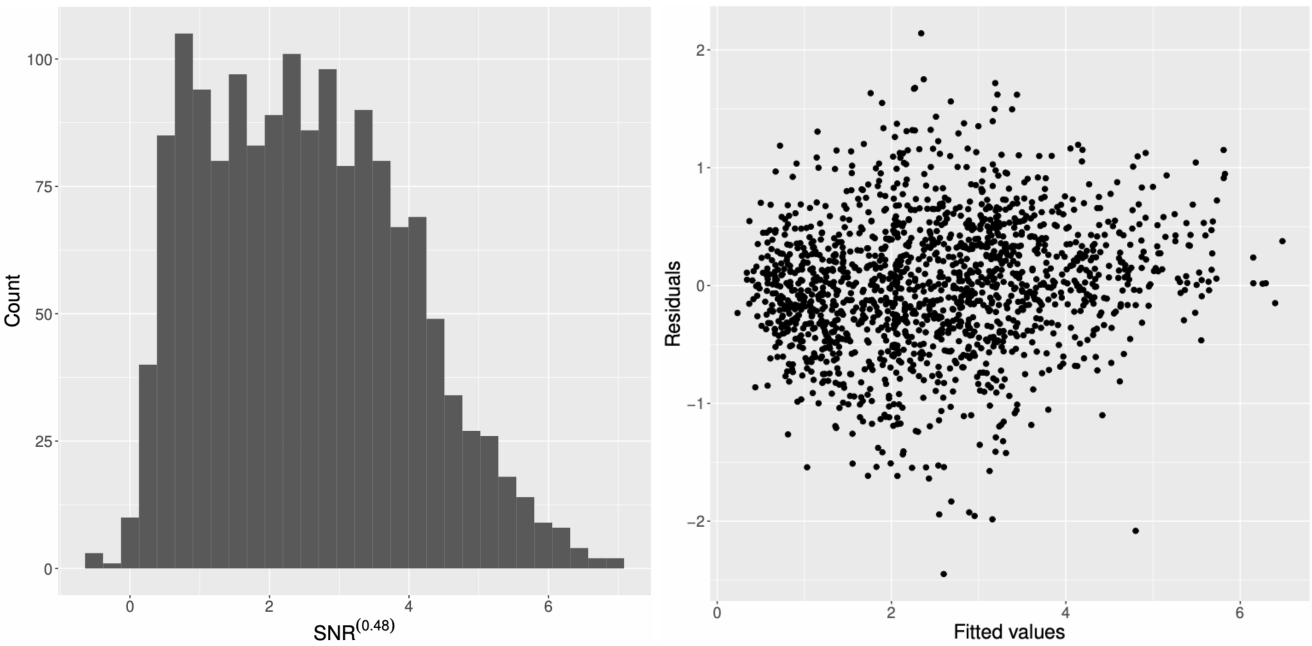

(a) Transformed Response.

(b) Fitted vs Residual Values.

Figure S.5. Transformed Data: a) After box-cox transformation of the SSVEP response, the previously right-skewed distribution is now normal. **b)** Data after normalisation via the box-cox transform.

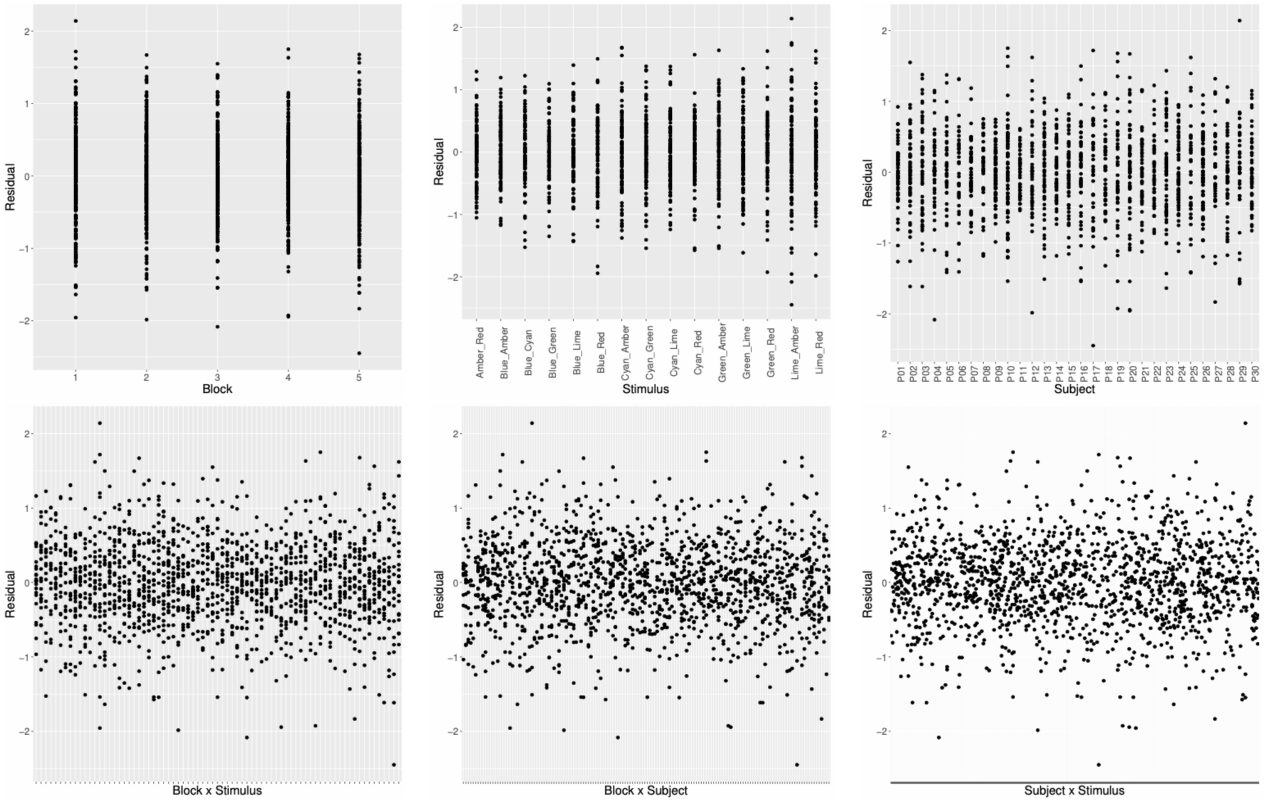

Figure S.6. Plot of the residuals against all effects in model S.11 of transformed data.

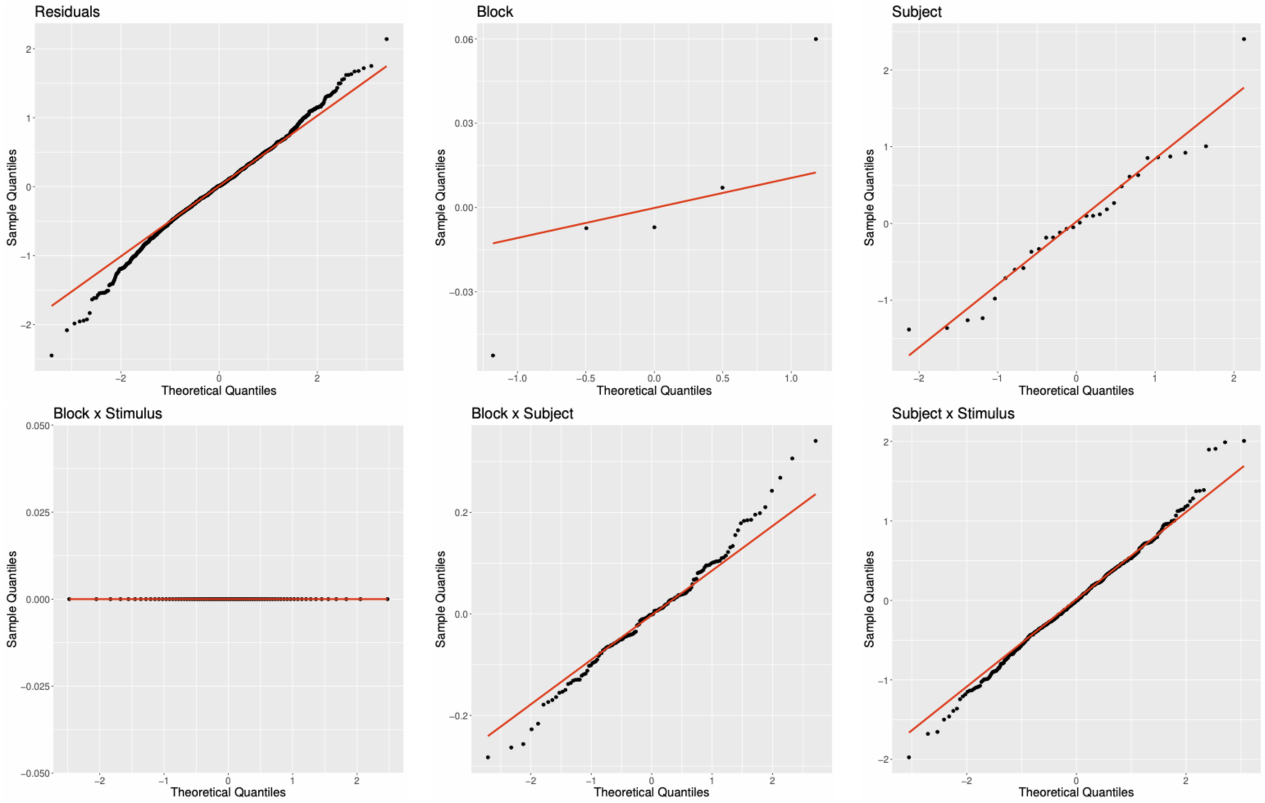

Figure S.7. Quantile-quantile plots of the residuals and the random effects for model S.11 of transformed data. Note that the qq-plot for the block-stimulus interaction is flat because the corresponding variance is essentially estimated as zero.

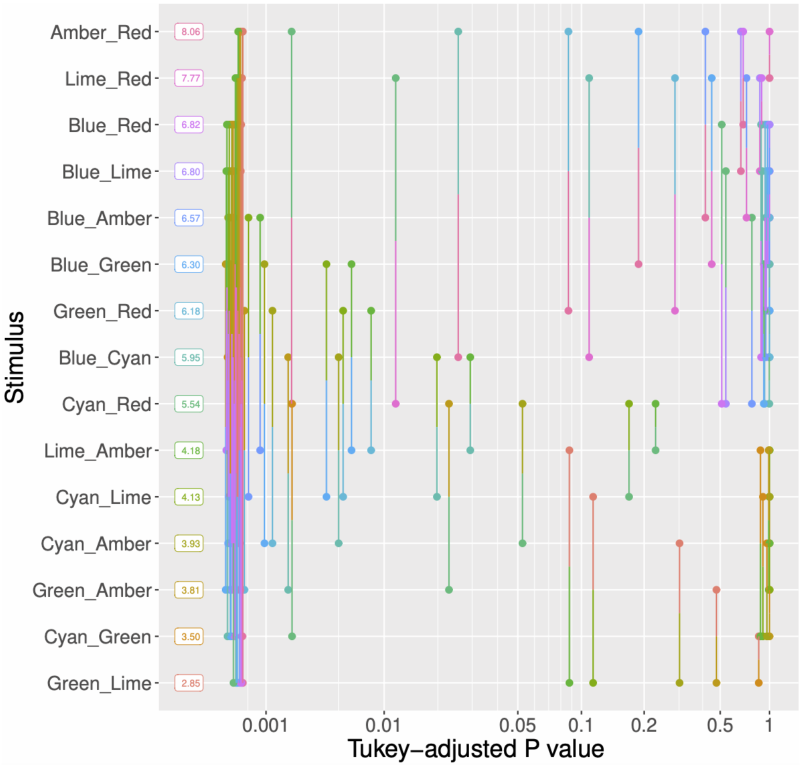

Figure S.8. Pairwise P-value plot of stimulus contrasts.

S.4 Extended Electroencephalography Results

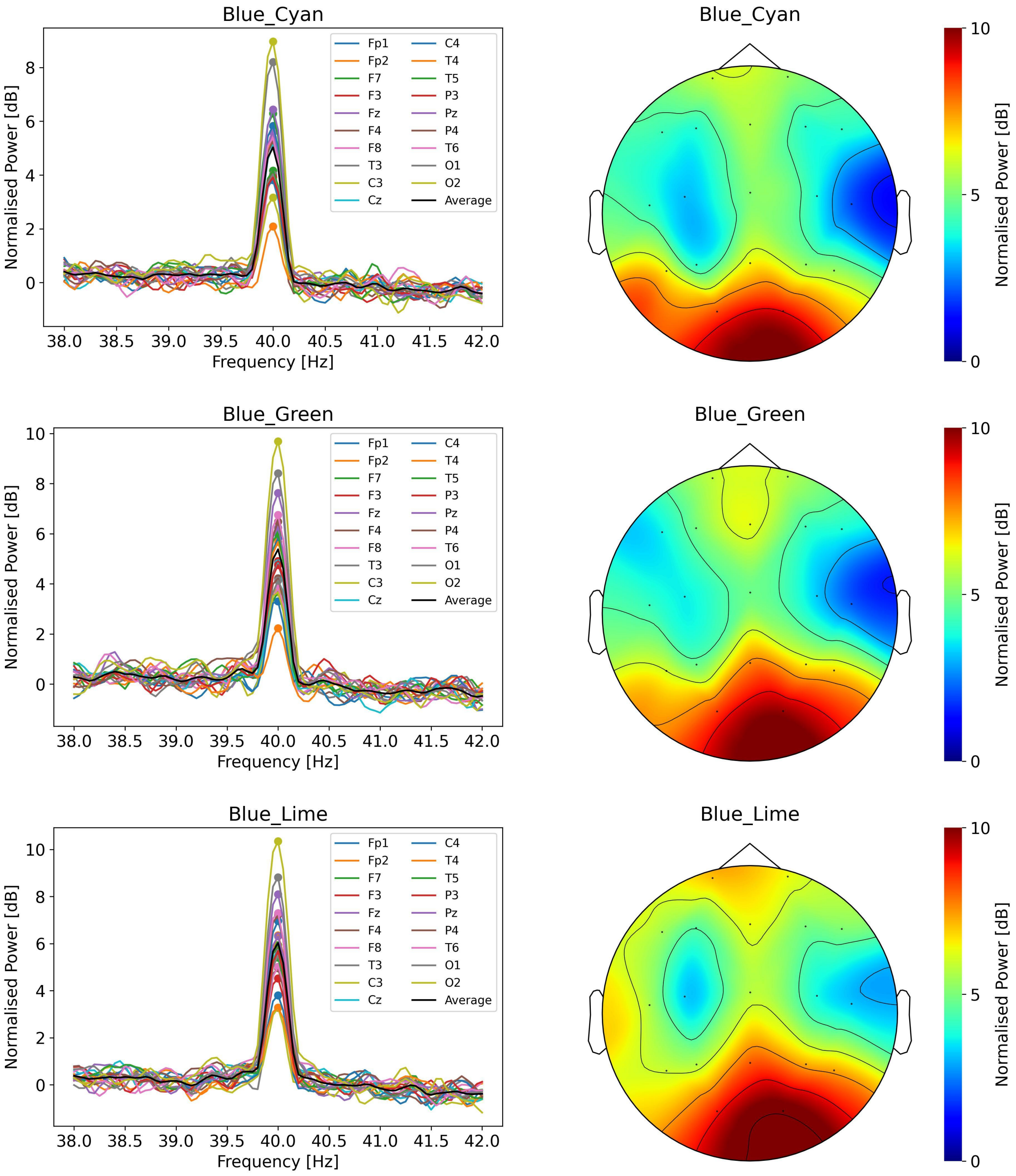

Figure S.9. Topographic Distribution of 40 Hz Signal: The [38;42] Hz band of the power spectral density estimate normalised to the average [38;39] Hz and [41;42] Hz bands are shown in the left panels, and the 40 Hz values (i.e. the peaks indicated by a dot in the left panels) are shown across the 19 EEG channels as topographic maps in the right panels. All plots are created from grand averages across repetitions and subjects. The top row shows the Blue/Cyan chromatic flicker stimulus, the middle row shows the Blue/Green chromatic flicker stimulus, and the bottom row shows the Blue/Lime chromatic flicker stimulus.

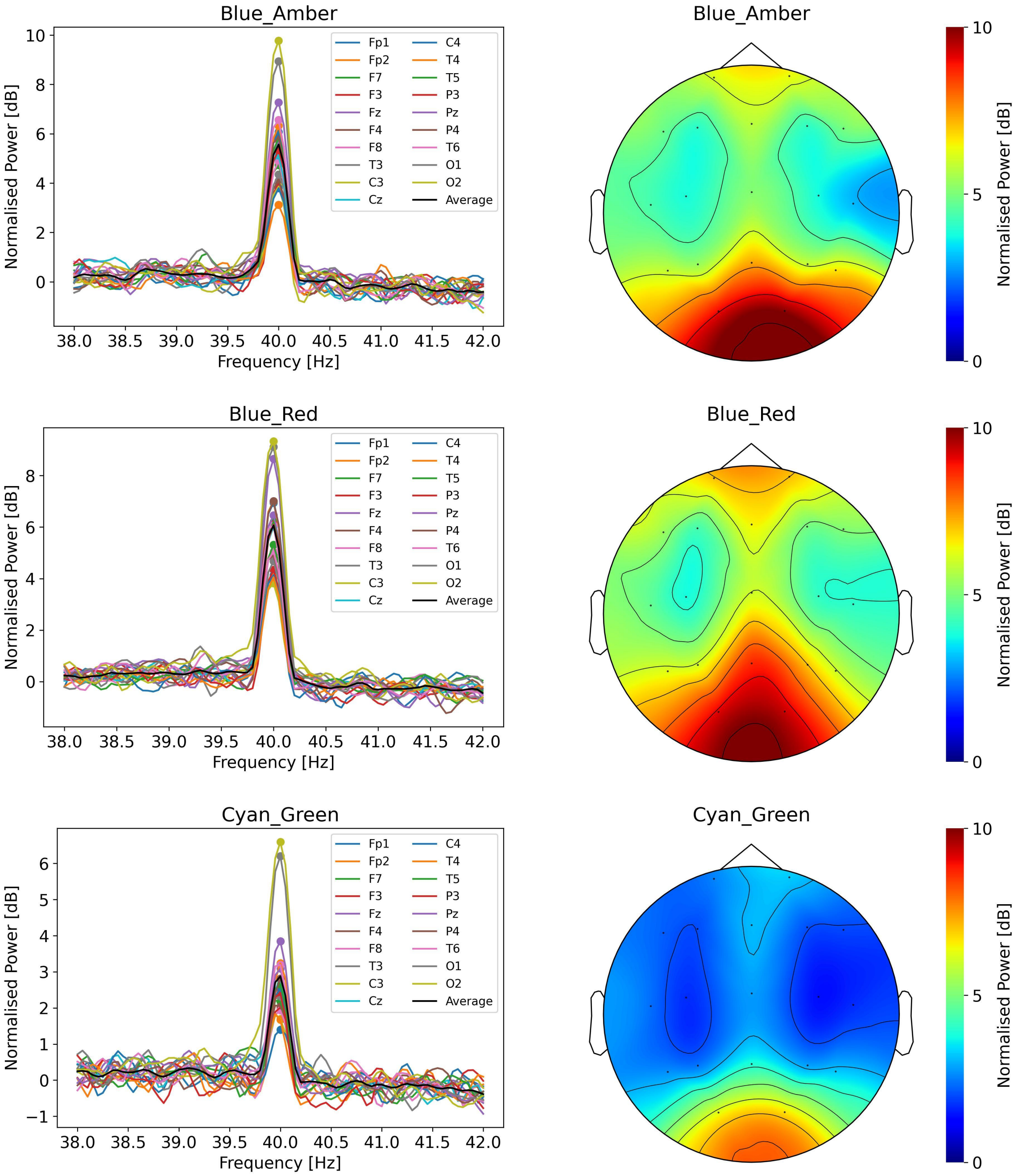

Figure S.10. Topographic Distribution of 40 Hz Signal: The [38;42] Hz band of the power spectral density estimate normalised to the average [38;39] Hz and [41;42] Hz bands are shown in the left panels, and the 40 Hz values (i.e. the peaks indicated by a dot in the left panels) are shown across the 19 EEG channels as topographic maps in the right panels. All plots are created from grand averages across repetitions and subjects. The top row shows the Blue/Amber chromatic flicker stimulus, the middle row shows the Blue/Red chromatic flicker stimulus, and the bottom row shows the Cyan/Green chromatic flicker stimulus

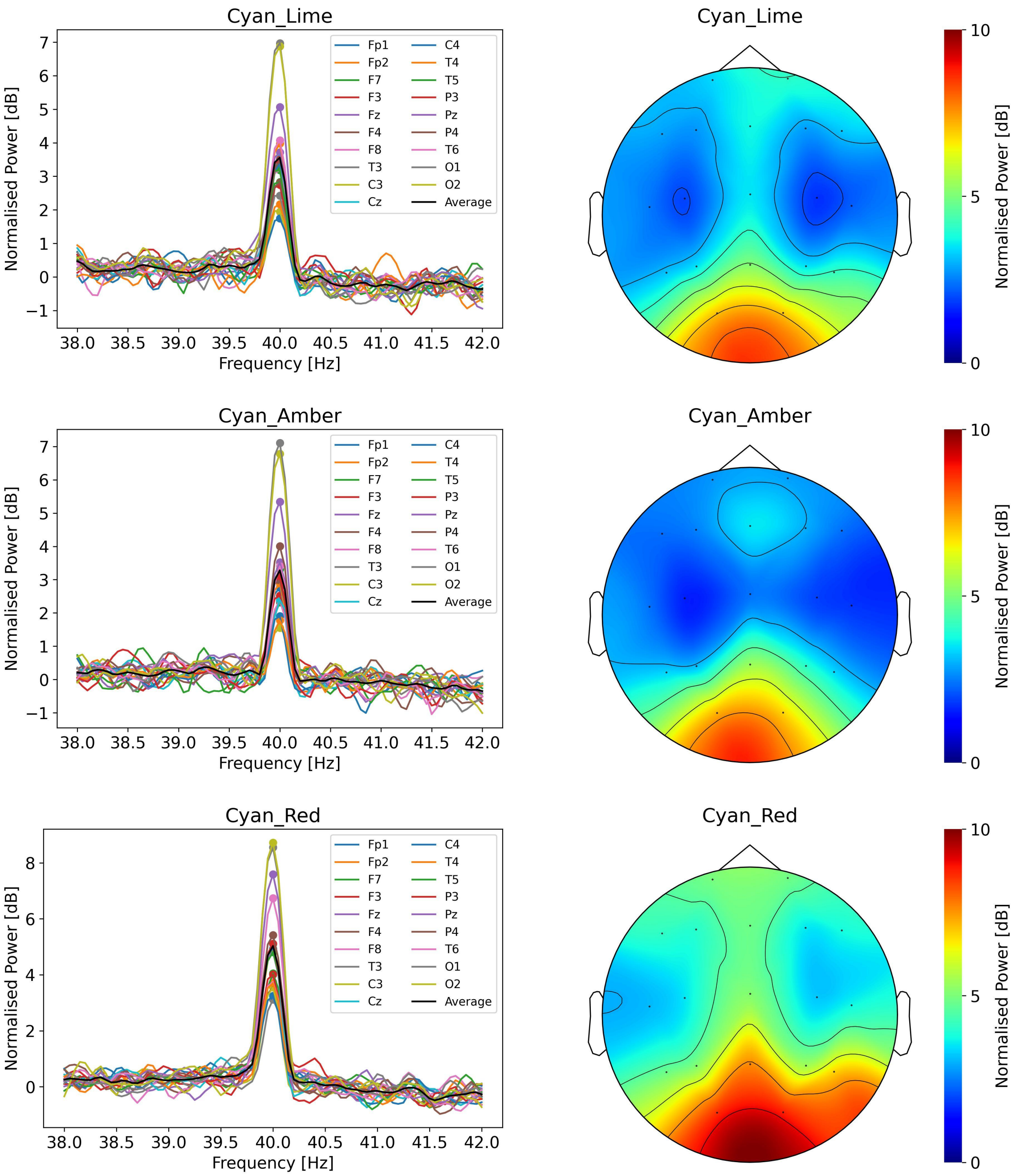

Figure S.11. Topographic Distribution of 40 Hz Signal: The [38;42] Hz band of the power spectral density estimate normalised to the average [38;39] Hz and [41;42] Hz bands are shown in the left panels, and the 40 Hz values (i.e. the peaks indicated by a dot in the left panels) are shown across the 19 EEG channels as topographic maps in the right panels. All plots are created from grand averages across repetitions and subjects. The top row shows the Cyan/Lime chromatic flicker stimulus, the middle row shows the Cyan/Amber chromatic flicker stimulus, and the bottom row shows the Cyan/Red chromatic flicker stimulus

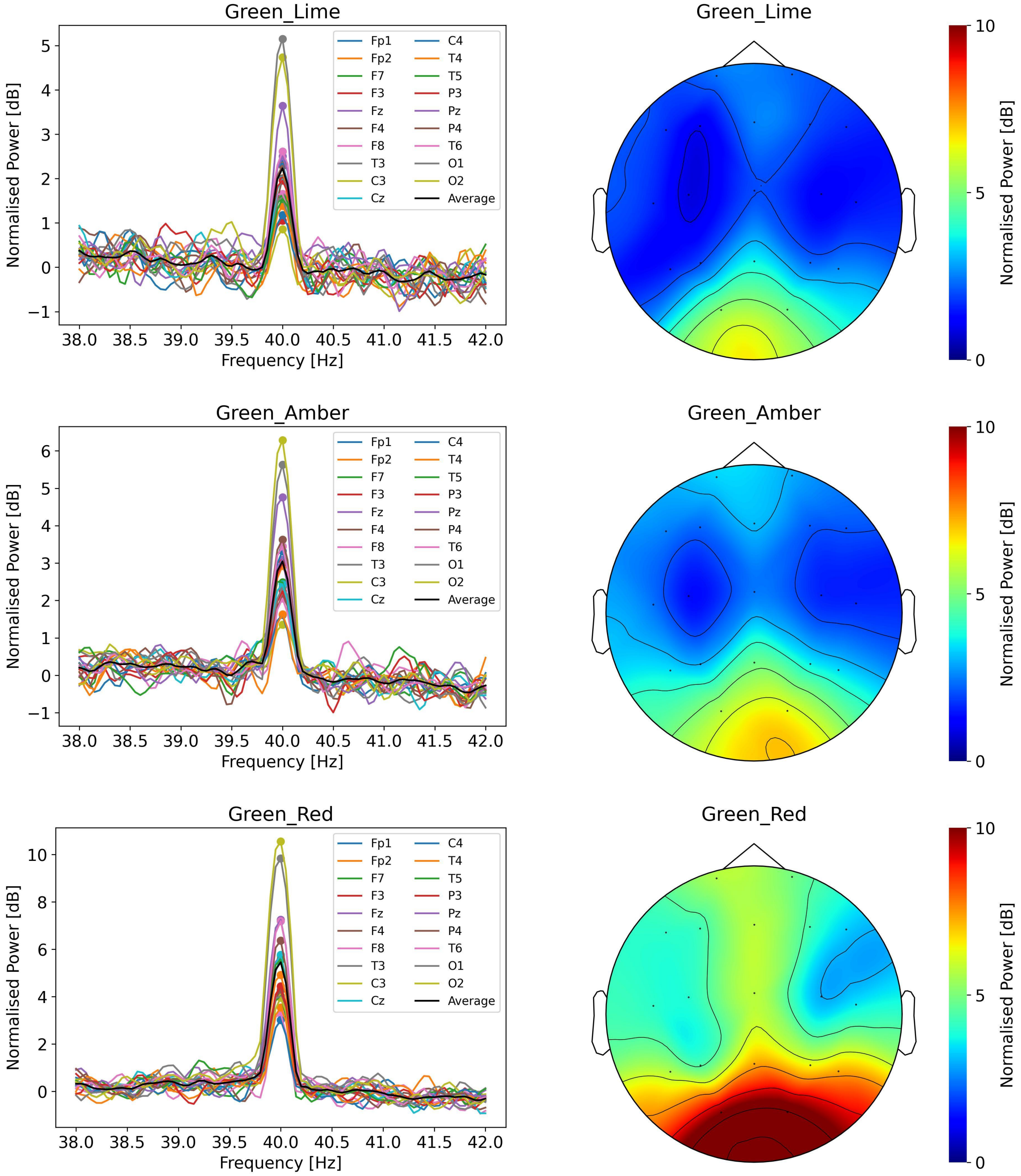

Figure S.12. **Topographic Distribution of 40 Hz Signal:** The [38;42] Hz band of the power spectral density estimate normalised to the average [38;39] Hz and [41;42] Hz bands are shown in the left panels, and the 40 Hz values (i.e. the peaks indicated by a dot in the left panels) are shown across the 19 EEG channels as topographic maps in the right panels. All plots are created from grand averages across repetitions and subjects. The top row shows the Green/Lime chromatic flicker stimulus, the middle row shows the Green/Amber chromatic flicker stimulus, and the bottom row shows the Green/Red chromatic flicker stimulus

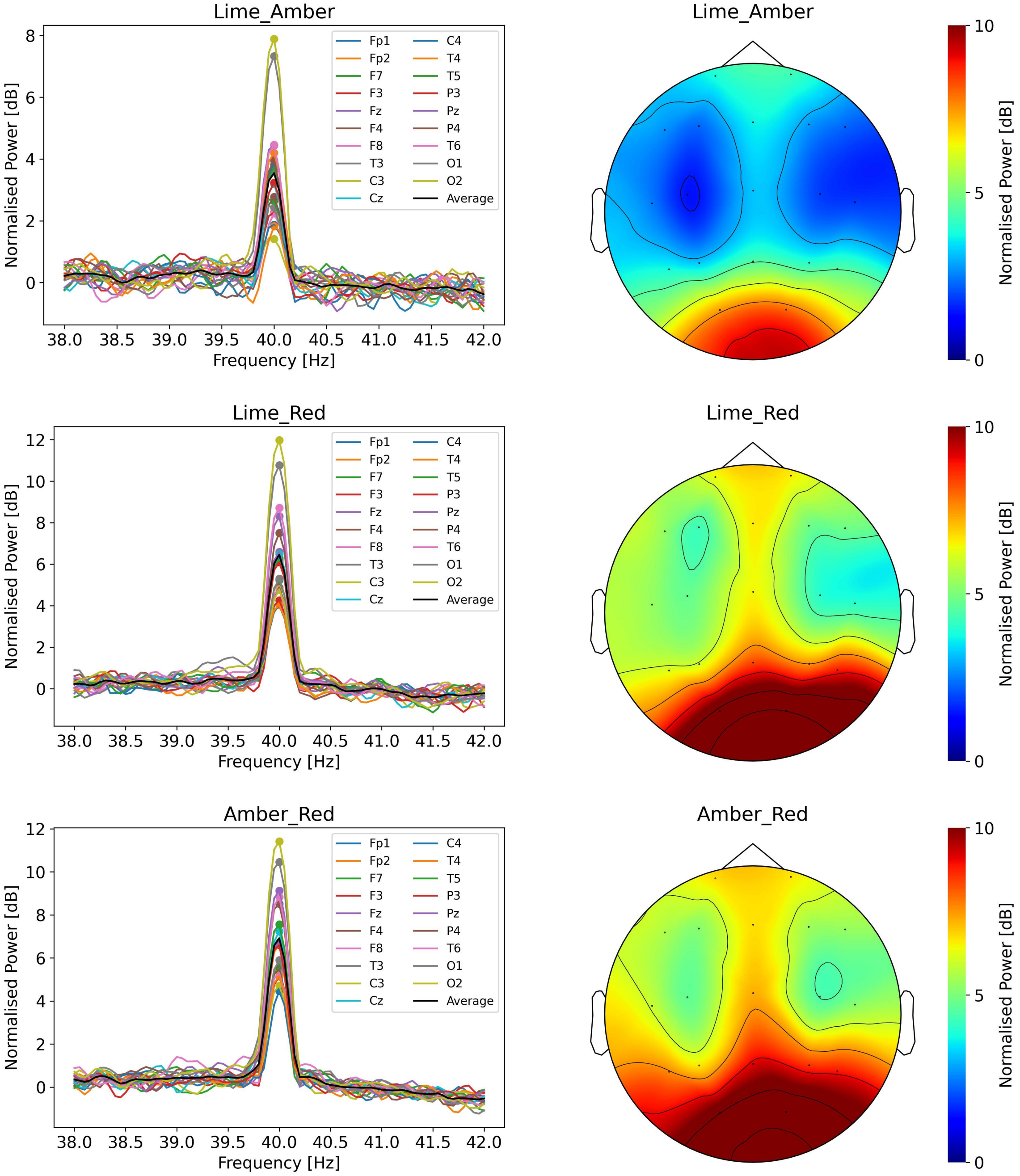

Figure S.13. Topographic Distribution of 40 Hz Signal: The [38;42] Hz band of the power spectral density estimate normalised to the average [38;39] Hz and [41;42] Hz bands are shown in the left panels, and the 40 Hz values (i.e. the peaks indicated by a dot in the left panels) are shown across the 19 EEG channels as topographic maps in the right panels. All plots are created from grand averages across repetitions and subjects. The top row shows the Lime/Amber chromatic flicker stimulus, the middle row shows the Lime/Red chromatic flicker stimulus, and the bottom row shows the Amber/Red chromatic flicker stimulus
